# Association between body temperature and all-cause mortality in patients with sepsis: analysis of the MIMIC-IV database

**DOI:** 10.1186/s40001-024-02219-2

**Published:** 2024-12-26

**Authors:** Yunuo Zhao, Bo Zhang

**Affiliations:** 1 Department of Biotherapy, Cancer Center and State Key Laboratory of Biotherapy, West China Hospital, Sichuan University, Chengdu, Sichuan China; 2 Department of Critical Care Medicine, West China Hospital, Sichuan University, Chengdu, Sichuan China

**Keywords:** Critical care, Abnormal body temperature, Sepsis, 28-day mortality

## Abstract

**Background:**

Abnormal body temperature (fever or hypothermia) is a critical symptom in sepsis and is strongly associated with clinical prognosis and disease progression. Given the duality and variability of body temperature fluctuations throughout the disease course, further research is essential to refine clinical strategies for temperature management in sepsis patients.

**Methods:**

We extracted clinical data of sepsis patients from the MIMIC-IV database. A restricted cubic spline (RCS) curve was employed to describe the non-linear relationship between body temperature and clinical outcomes. Based on peak temperature within the first 24 h after admission, patients were categorized into three groups: < 36 °C, 36–38 °C, and > 38 °C. We subsequently matched patients one-to-one into three cohorts using a pairwise propensity score matching (PSM) approach. Alongside clinical data, we conducted log-rank and McNemar tests, and established multiple models, including multiple Cox regression, overlap-weighted (OW) adjusted Cox regression, multiple logistic regression, and OW-adjusted multiple logistic regression, to investigate the impact of temperature on clinical outcomes.

**Results:**

A total of 35,499 sepsis patients were included in my study: 311 with a temperature below 36 °C, 27,538 with a temperature between 36 and 38 °C, and 7650 with a temperature above 38 °C. The RCS analysis revealed a non-linear, U-shaped relationship between body temperature and 28-day, ICU, and in-hospital mortality. Patients with hypothermia had significantly higher 28-day mortality (54.34% vs. 19.28%), ICU mortality (44.37% vs. 12.89%), and in-hospital mortality (49.20% vs. 17.46%) compared to those with hyperthermia. Among patients younger than 65 years, hyperthermia was a protective factor against 28-day mortality relative to normal body temperature, while the opposite was observed in patients aged 65 and older. This trend was consistent in the analysis of ICU and in-hospital mortality.

**Conclusions:**

Among sepsis patients admitted to the ICU, a peak temperature below 36 °C within the first 24 h of admission was associated with higher 28-day mortality. However, no significant difference in clinical prognosis was observed between normothermic and hyperthermic patients.

**Supplementary Information:**

The online version contains supplementary material available at 10.1186/s40001-024-02219-2.

## Background

Sepsis is a clinical syndrome characterized by a high mortality rate, resulting from a dysregulated host response and life-threatening organ dysfunction [1, 2]. Its primary clinical manifestations include chills, palpitations, shortness of breath, and altered mental status [3]. Among these, abnormal body temperature (fever or hypothermia) is not only a key symptom of sepsis but also a critical indicator for assessing the severity and prognosis of the condition [4, 5]. As a vital sign parameter that is easily obtainable and holds significant diagnostic value in clinical practice, it is widely recognized by researchers [6, 7].

Fever typically arises from the body's immune response to infection, which suppresses pathogen growth and enhances immune function, though it may also increase metabolic demands and cardiovascular strain [8]. The need for hypothermia therapy in febrile sepsis remains a clinical controversy. Conversely, hypothermia is often linked to higher mortality rates, especially among older patients or those with weakened immune systems, as it may signify an inadequate immune response or an advanced disease stage [9]. Therefore, temperature management is a crucial aspect of sepsis treatment: antipyretics and physical cooling methods can alleviate hyperthermia symptoms, while warming devices can be employed to address hypothermia, ultimately aiming to reduce complications and improve patient outcomes. Given the dual and heterogeneous nature of temperature abnormalities in sepsis progression, further studies are required to refine clinical strategies for temperature control in septic patients.

Patients with sepsis from the MIMIC-IV database were categorized based on body temperature into three groups: hypothermia group (< 36 °C), normal temperature group (36–38 °C), and hyperthermia group (> 38 °C). Combined with other clinical data, the objective is to explore the relationship between abnormal body temperature and 28-day, ICU, and in-hospital mortality in patients with sepsis.

## Methods

### Study design

We conducted a retrospective cohort study using a large database, the Medical Information Mart for Intensive Care IV (v3.0). The MIMIC-IV (v3.0) database is a large publicly available dataset containing de-identified health information for patients admitted to the Beth Israel Deaconess Medical Center in Boston for intensive-care unit from 2008 to 2022.

The author obtained a Collaborative Institutional Training Initiative (CITI program) Certificate (Record ID: 61575775), which enabled access to the database and data extraction. The study complied with the Reporting of Studies Conducted using Observational Routinely Collected Health Data (RECORD) statement.

### Selection of participants

Based on the Sepsis-3 criteria, this study included patients from the MIMIC-IV database who were diagnosed with sepsis, defined as those with documented or suspected acute infection and Sequential Organ Failure Assessment (SOFA) score ≥ 2. We initially enrolled 41,296 patients, excluding those under 18 years of age or with an ICU stay of less than 24 h. Patients lacking complete data on maximum temperature within the first 24 h of ICU admission were also excluded, resulting in a final study cohort of 35,499 patients. I divided the patients into three groups according to their maximum body temperature within 24 h after admission: hypothermia group (< 36 °C), normal temperature group (36–38 °C), and hyperthermia group (> 38 °C), and formed three cohorts: cohort 1 (< 36 °C and 36–38 °C), cohort 2 (< 36 °C and > 38 °C), cohort 3 (> 38 °C and 36–38 °C).

### Variable extraction and outcome

We extracted the baseline characteristics of the included patients within 24 h of ICU admission using Structured Query Language (SQL) with PostgreSQL (version 16), including age, gender, weight, and illness severity indicators (SOFA score and Charlson score). Data on mechanical ventilation, sedatives, vasopressors, albumin, and comorbidities such as heart failure (HF), atrial fibrillation (AFIB), renal disease, liver disease, chronic obstructive pulmonary disease (COPD), coronary artery disease (CAD), stroke, and malignant tumor were also obtained, with comorbidities identified via recorded ICD-9 codes. Vital signs measured for the first time within 24 h of admission included mean arterial pressure (MAP) and heart rate. Laboratory variables, including white blood cell (WBC) count, hemoglobin, platelet count, sodium, potassium, bicarbonate, chloride, blood urea nitrogen (BUN), lactate, creatinine, pH, partial pressure of oxygen (PO_2_), and partial pressure of carbon dioxide (PCO_2_), were measured within the first 24 h of ICU admission. The primary outcome was 28-day mortality. Secondary outcomes included in-hospital and ICU mortality.

### Statistical analysis

Values are presented as the medians (interquartile ranges) or means (standard deviations) for continuous variables. Categorical variables are presented as total numbers and percentages. Group comparisons were performed using the *χ*^2^ test or Fisher’s exact test for categorical variables, and Student’s *t*-test or the Mann–Whitney *U* test for continuous variables, as appropriate.

We employed Restricted Cubic Splines (RCS) to illustrate the non-linear relationship between patient temperature—specifically, the maximum and minimum body temperatures recorded within 24 h of ICU admission—and the study outcomes. To ensure the robustness of the statistical results, we used pairwise propensity score matching (PSM) and overlap-weighted (OW) based on this to adjust for each included covariate to ensure relative balance [10, 11]. The study identified 3 pairwise 1:1 propensity score–matched cohorts: cohort 1 (< 36 °C group and 36–38 °C group), cohort 2 (< 36 °C group and > 38 °C group), cohort 3 (36–38 °C group and > 38 °C group). Kaplan–Meier (K-M) survival curves were plotted for different cohorts to show 28-day, ICU, and in-hospital survival for patients with sepsis and were compared using log-rank tests.

We used multiple regression to analyze the association between body temperature and outcomes. Baseline variables considered clinically relevant entered the multiple Cox regression and multiple logistic regression as covariates. Using the estimated propensity score as the weight, the OW model is established. Standardized mean difference (SMD) was calculated to evaluate the effectiveness of PSM and OW. Then the matching queue and the weighted queue are tested by multiple Cox regression and multiple logistic regression model. In addition, we constructed a double-robust model that combined a regression model for outcomes and a propensity score model for treatments to further assess the robustness of the model results. Finally, we also performed a subgroup analysis to explore the relationship between abnormal body temperature and clinical outcomes in different subgroups of patients, including age, SOFA score, HF, CAD, albumin use and malignancy. To avoid bias induced by missing data, the analysis of the outcome was duplicated after multiple imputations. Additionally, variables with missing values exceeding 40% were excluded from the analytical models as covariates.

All statistical analyses were performed using RStudio and *p* < 0.05 was considered statistically significant. We employed the survival, car, MatchIt, and forestplot packages to develop models and conduct data analysis.

## Results

### Baseline characteristics

**Fig. 1:**
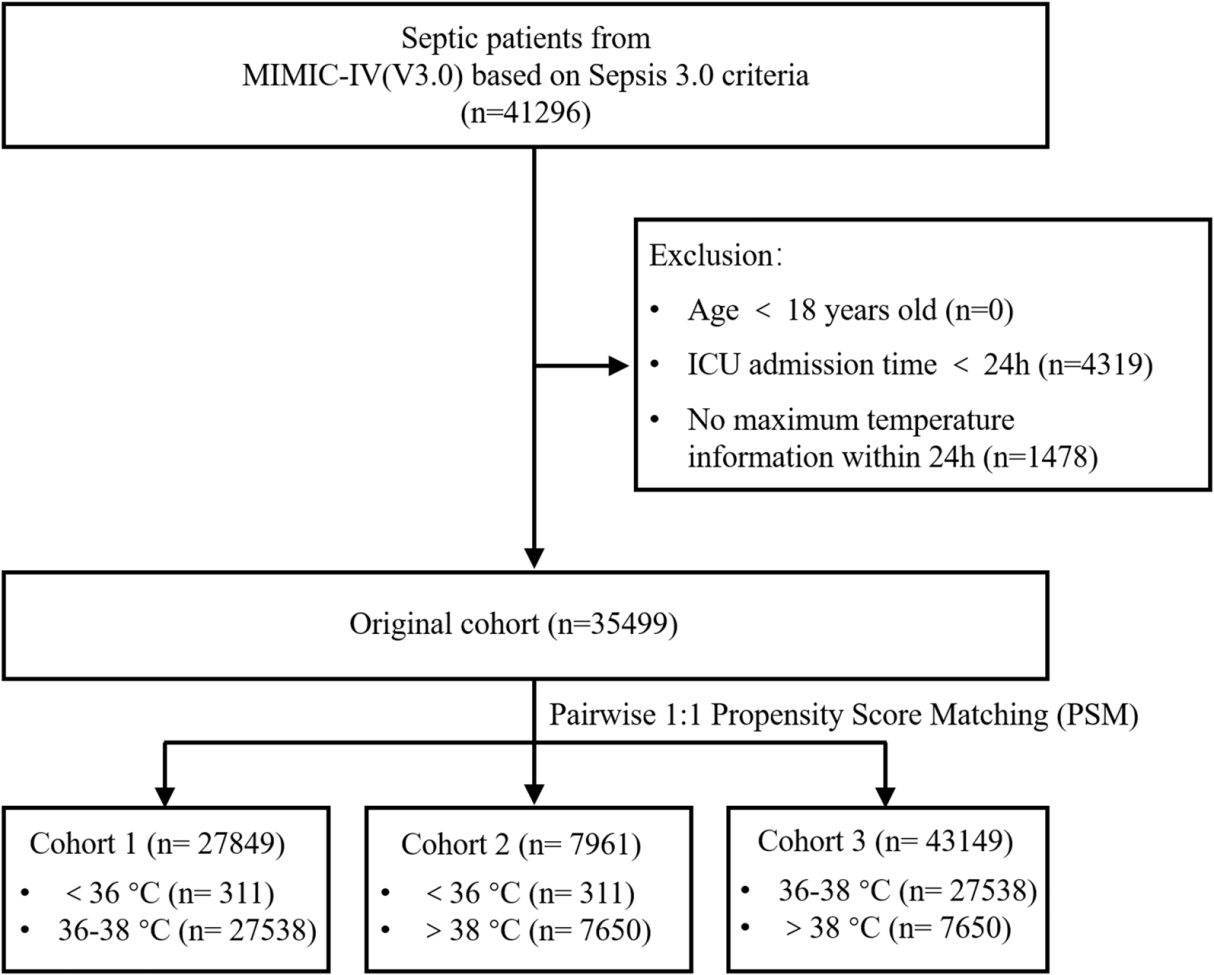
The detailed process of data extraction

Using the Sepsis 3.0 criteria, 41,296 patients with sepsis were identified in the database. After excluding patients younger than 18 years, those with an ICU stay of less than 24 h, and individuals with missing maximum body temperature data, the final study cohort consisted of 35,499 patients (Fig. 1). Patients were classified into three groups based on body temperature, and their baseline characteristics are summarized in Table S1. A pairwise propensity score matching (PSM) approach was then employed, matching patients one-to-one into three cohorts, with their characteristics detailed in Tables 1, 2, 3. Additionally, we have included in the supplementary material the patient’s hadm ID, stay ID, subject ID, and the time at which they met the Sepsis-3 diagnostic criteria for reference.

**Table 1:** Baseline characteristics before and after propensity score matching of cohort 1

	Before matching				After matching			
	Overall (*N* = 27,849)	< 36℃ (*N* = 311)	36 ℃–38 ℃ (*N* = 27,538)	SMD	Overall (*N* = 592)	< 36 ℃ (*N* = 298)	36 ℃–38 ℃ (*N* = 298)	SMD
Age	67.34 (15.39)	66.00 (16.58)	67.36 (15.37)	0.085	65.62 (15.78)	66.56 (16.40)	64.68 (15.10)	0.12
Gender (female)	12,045 (43.25%)	123 (39.55%)	11,922 (43.29%)	0.076	227 (38.34%)	120 (40.54%)	107 (36.15%)	0.09
Weight	81.51 (24.01)	80.06 (20.04)	81.53 (24.05)	0.066	79.42 (20.38)	79.85 (20.12)	78.99 (20.66)	0.043
SOFA score	**5.96 (3.46)**	**8.58 (3.88)**	**5.93 (3.45)**	**0.722**	8.41 (4.06)	8.30 (3.71)	8.52 (4.38)	0.053
Charlson score	5.71 (2.95)	5.42 (2.90)	5.71 (2.95)	0.101	5.45 (2.87)	5.50 (2.87)	5.40 (2.88)	0.034
Interventions (boolean for 1st 24 h)								
Mechanical ventilation use (YES)	**13,222 (47.48%)**	**232 (74.60%)**	**12,990 (47.17%)**	**0.586**	439 (74.16%)	217 (73.31%)	222 (75.00%)	0.039
Vasopressor use (YES)	**11,493 (41.27%)**	**200 (64.31%)**	**11,293 (41.01%)**	**0.48**	390 (65.88%)	186 (62.84%)	204 (68.92%)	0.129
Sedative use (YES)	**13,043 (46.83%)**	**207 (66.56%)**	**12,836 (46.61%)**	**0.411**	400 (67.57%)	193 (65.20%)	207 (69.93%)	0.101
Albumin use (YES)	4194 (15.06%)	50 (16.08%)	4144 (15.05%)	0.028	102 (17.23%)	48 (16.22%)	54 (18.24%)	0.054
Comorbidities (boolean)								
HF (YES)	9473 (34.02%)	117 (37.62%)	9356 (33.97%)	0.076	221 (37.33%)	113 (38.18%)	108 (36.49%)	0.035
AFIB (YES)	**4651 (16.70%)**	**83 (26.69%)**	**4568 (16.59%)**	**0.247**	148 (25.00%)	79 (26.69%)	69 (23.31%)	0.078
Renal (YES)	7824 (28.09%)	94 (30.23%)	7730 (28.07%)	0.047	180 (30.41%)	92 (31.08%)	88 (29.73%)	0.029
Liver (YES)	**2796 (10.04%)**	**20 (6.43%)**	**2776 (10.08%)**	**0.133**	37 (6.25%)	19 (6.42%)	18 (6.08%)	0.014
COPD (YES)	4756 (17.08%)	51 (16.40%)	4705 (17.09%)	0.018	100 (16.89%)	49 (16.55%)	51 (17.23%)	0.018
CAD (YES)	9089 (32.64%)	108 (34.73%)	8981 (32.61%)	0.045	212 (35.81%)	105 (35.47%)	107 (36.15%)	0.014
Stroke (YES)	2884 (10.36%)	31 (9.97%)	2853 (10.36%)	0.013	69 (11.66%)	31 (10.47%)	38 (12.84%)	0.074
Malignancy (YES)	**4596 (16.50%)**	**30 (9.65%)**	**4566 (16.58%)**	**0.207**	63 (10.64%)	30 (10.14%)	33 (11.15%)	0.033
Vital signs (1st 24 h)								
MAP	82.05 (19.11)	82.17 (20.14)	82.05 (19.10)	0.006	83.00 (19.59)	82.36 (20.18)	83.64 (19.00)	0.065
Heart rate	**89.71 (20.24)**	**84.09 (20.81)**	**89.78 (20.22)**	**0.277**	85.06 (20.48)	84.14 (20.99)	85.99 (19.96)	0.091
Laboratory tests (1st 24 h)								
WBC	**13.22 (10.42)**	**14.83 (8.78)**	**13.20 (10.44)**	**0.169**	15.32 (16.00)	14.66 (8.75)	15.98 (20.87)	0.082
Hemoglobin	**10.19 (2.23)**	**10.93 (2.74)**	**10.18 (2.23)**	**0.298**	10.85 (2.58)	10.89 (2.69)	10.81 (2.47)	0.028
Platelet	**203.57 (115.67)**	**213.66 (109.25)**	**203.46 (115.74)**	**0.091**	215.48 (113.69)	213.01 (109.84)	217.95 (117.55)	0.043
Sodium	137.69 (7.95)	138.05 (5.80)	137.69 (7.97)	0.052	137.89 (5.93)	137.99 (5.74)	137.79 (6.12)	0.034
Potassium	4.30 (0.84)	4.39 (0.99)	4.30 (0.84)	0.093	4.38 (0.92)	4.40 (0.98)	4.36 (0.87)	0.035
Bicarbonate	**22.62 (5.26)**	**19.91 (5.93)**	**22.65 (5.25)**	**0.49**	20.24 (5.68)	20.18 (5.76)	20.31 (5.62)	0.023
Chloride	103.34 (7.17)	104.00 (6.97)	103.33 (7.17)	0.095	104.10 (7.12)	104.19 (6.93)	104.01 (7.31)	0.025
BUN	**32.31 (26.02)**	**38.40 (30.88)**	**32.24 (25.95)**	**0.216**	38.38 (32.20)	38.53 (31.35)	38.22 (33.07)	0.01
Lactate	**2.28 (1.88)**	**4.08 (3.73)**	**2.26 (1.84)**	**0.621**	3.66 (3.26)	3.71 (3.06)	3.61 (3.45)	0.031
Creatinine	**1.71 (1.77)**	**2.20 (2.23)**	**1.70 (1.76)**	**0.249**	2.20 (2.24)	2.16 (2.22)	2.23 (2.26)	0.031
pH	**7.36 (0.10)**	**7.28 (0.14)**	**7.36 (0.10)**	**0.701**	7.29 (0.13)	7.29 (0.13)	7.29 (0.13)	0.011
PO2	163.51 (117.07)	158.70 (121.79)	163.57 (117.01)	0.041	161.82 (122.17)	160.49 (122.32)	163.15 (122.20)	0.022
PCO2	42.98 (13.49)	43.82 (15.05)	42.97 (13.47)	0.06	43.66 (15.16)	43.50 (14.65)	43.82 (15.68)	0.021
Outcomes (boolean)								
28-day mortality (Death)	**6014 (21.60%)**	**169 (54.34%)**	**5845 (21.23%)**	**0.727**	**243 (41.05%)**	**157 (53.04%)**	**86 (29.05%)**	**0.503**
ICU mortality (Death)	**3324 (11.94%)**	**138 (44.37%)**	**3186 (11.57%)**	**0.785**	**185 (31.25%)**	**126 (42.57%)**	**59 (19.93%)**	**0.504**
In-hospital mortality (Death)	**4926 (17.69%)**	**153 (49.20%)**	**4773 (17.33%)**	**0.719**	**215 (36.32%)**	**141 (47.64%)**	**74 (25.00%)**	**0.484**
Length of stay (days)								
ICU length of stay	**5.74 (7.30)**	**6.51 (7.42)**	**5.73 (7.30)**	**0.106**	7.09 (8.95)	6.55 (7.48)	7.63 (10.19)	0.122
Hospital length of stay	**12.73 (14.10)**	**10.47 (12.03)**	**12.75 (14.12)**	**0.174**	**12.92 (19.53)**	**10.69 (12.20)**	**15.15 (24.60)**	**0.23**

Values are presented as mean (standard deviation) for continuous variables and number (percentage) for categorical variables. Variables in bold have *p* < 0.05

**Table 2:** Baseline characteristics before and after propensity score matching of cohort 2

	Before matching				After matching			
	Overall (*N* = 7961)	< 36 ℃ (*N* = 311)	> 38 ℃ (*N* = 7650)	SMD	Overall (*N* = 594)	< 36 ℃ (*N* = 297)	> 38 ℃ (*N* = 297)	SMD
Age	**61.49 (16.95)**	**66.00 (16.58)**	**61.30 (16.94)**	**0.281**	66.09 (16.46)	65.89 (16.60)	66.29 (16.34)	0.024
Gender (Female)	3047 (38.27%)	123 (39.55%)	2924 (38.22%)	0.027	242 (40.74%)	117 (39.39%)	125 (42.09%)	0.055
Weight	**84.27 (24.23)**	**80.06 (20.04)**	**84.44 (24.37)**	**0.196**	79.06 (20.17)	80.01 (20.09)	78.10 (20.24)	0.095
SOFA score	**6.36 (3.58)**	**8.58 (3.88)**	**6.27 (3.54)**	**0.623**	8.46 (4.00)	8.42 (3.82)	8.51 (4.17)	0.022
Charlson score	**4.75 (3.06)**	**5.42 (2.90)**	**4.72 (3.06)**	**0.234**	5.48 (2.99)	5.40 (2.89)	5.56 (3.09)	0.053
Interventions (boolean for 1st 24 h)								
Mechanical ventilation use (YES)	**5119 (64.30%)**	**232 (74.60%)**	**4887 (63.88%)**	**0.234**	436 (73.40%)	220 (74.07%)	216 (72.73%)	0.03
Vasopressor use (YES)	**3657 (45.94%)**	**200 (64.31%)**	**3457 (45.19%)**	**0.391**	377 (63.47%)	189 (63.64%)	188 (63.30%)	0.007
Sedative use (YES)	**4847 (60.88%)**	**207 (66.56%)**	**4640 (60.65%)**	**0.123**	398 (67.00%)	197 (66.33%)	201 (67.68%)	0.029
Albumin use (YES)	**886 (11.13%)**	**50 (16.08%)**	**836 (10.93%)**	**0.151**	97 (16.33%)	48 (16.16%)	49 (16.50%)	0.009
Comorbidities (boolean)								
HF (YES)	**2066 (25.95%)**	**117 (37.62%)**	**1949 (25.48%)**	**0.264**	230 (38.72%)	110 (37.04%)	120 (40.40%)	0.069
AFIB (YES)	**1142 (14.34%)**	**83 (26.69%)**	**1059 (13.84%)**	**0.324**	146 (24.58%)	79 (26.60%)	67 (22.56%)	0.094
Renal (YES)	**1610 (20.22%)**	**94 (30.23%)**	**1516 (19.82%)**	**0.242**	182 (30.64%)	87 (29.29%)	95 (31.99%)	0.058
Liver (YES)	513 (6.44%)	20 (6.43%)	493 (6.44%)	0.001	38 (6.40%)	19 (6.40%)	19 (6.40%)	< 0.001
COPD (YES)	**973 (12.22%)**	**51 (16.40%)**	**922 (12.05%)**	**0.125**	107 (18.01%)	48 (16.16%)	59 (19.87%)	0.096
CAD (YES)	**1910 (23.99%)**	**108 (34.73%)**	**1802 (23.56%)**	**0.248**	225 (37.88%)	106 (35.69%)	119 (40.07%)	0.09
Stroke (YES)	1090 (13.69%)	31 (9.97%)	1059 (13.84%)	0.12	64 (10.77%)	31 (10.44%)	33 (11.11%)	0.022
Malignancy (YES)	**1222 (15.35%)**	**30 (9.65%)**	**1192 (15.58%)**	**0.179**	56 (9.43%)	30 (10.10%)	26 (8.75%)	0.046
Vital signs (1st 24 h)								
MAP	83.06 (19.54)	82.17 (20.14)	83.09 (19.51)	0.047	82.28 (20.13)	82.42 (19.74)	82.14 (20.55)	0.014
Heart rate	**97.94 (21.88)**	**84.09 (20.81)**	**98.50 (21.74)**	**0.678**	84.19 (20.63)	84.71 (20.88)	83.68 (20.39)	0.05
Laboratory tests (1st 24 h)								
WBC	**13.63 (12.23)**	**14.83 (8.78)**	**13.58 (12.35)**	**0.116**	14.95 (11.45)	14.72 (8.74)	15.18 (13.65)	0.04
Hemoglobin	**10.54 (2.31)**	**10.93 (2.74)**	**10.52 (2.29)**	**0.159**	10.91 (2.60)	10.95 (2.71)	10.88 (2.48)	0.028
Platelet	207.94 (118.33)	213.66 (109.25)	207.71 (118.68)	0.052	213.59 (112.30)	213.38 (108.20)	213.80 (116.44)	0.004
Sodium	138.23 (5.60)	138.05 (5.80)	138.24 (5.60)	0.033	138.07 (6.02)	138.04 (5.75)	138.10 (6.29)	0.01
Potassium	**4.18 (0.79)**	**4.39 (0.99)**	**4.17 (0.78)**	**0.249**	4.41 (0.95)	4.39 (0.99)	4.43 (0.91)	0.047
Bicarbonate	**22.40 (4.83)**	**19.91 (5.93)**	**22.50 (4.75)**	**0.483**	20.10 (5.54)	20.22 (5.74)	19.97 (5.34)	0.045
Chloride	104.19 (7.49)	104.00 (6.97)	104.19 (7.51)	0.026	104.20 (7.24)	104.04 (7.00)	104.37 (7.49)	0.046
BUN	**26.62 (20.97)**	**38.40 (30.88)**	**26.14 (20.33)**	**0.469**	38.34 (29.88)	37.08 (29.50)	39.61 (30.25)	0.085
Lactate	**2.34 (1.94)**	**4.08 (3.73)**	**2.27 (1.80)**	**0.619**	**3.57 (3.23)**	**3.81 (3.17)**	**3.33 (3.28)**	**0.149**
Creatinine	**1.52 (1.55)**	**2.20 (2.23)**	**1.49 (1.51)**	**0.374**	2.17 (2.16)	2.13 (2.21)	2.21 (2.11)	0.035
pH	**7.36 (0.10)**	**7.28 (0.14)**	**7.36 (0.10)**	**0.701**	7.29 (0.12)	7.29 (0.13)	7.29 (0.12)	0.016
PO2	159.05 (109.68)	158.70 (121.79)	159.07 (109.17)	0.003	160.42 (116.19)	160.36 (122.12)	160.48 (110.14)	0.001
PCO2	42.32 (13.06)	43.82 (15.05)	42.26 (12.97)	0.111	43.83 (15.78)	44.05 (14.84)	43.61 (16.69)	0.028
Outcomes (boolean)								
28-day mortality (Death)	**1644 (20.65%)**	**169 (54.34%)**	**1475 (19.28%)**	**0.78**	**241 (40.57%)**	**157 (52.86%)**	**84 (28.28%)**	**0.517**
ICU mortality (Death)	**1124 (14.12%)**	**138 (44.37%)**	**986 (12.89%)**	**0.743**	**194 (32.66%)**	**126 (42.42%)**	**68 (22.90%)**	**0.426**
In-hospital mortality (Death)	**1489 (18.70%)**	**153 (49.20%)**	**1336 (17.46%)**	**0.715**	**223 (37.54%)**	**141 (47.47%)**	**82 (27.61%)**	**0.419**
Length of stay (days)								
ICU length of stay	**7.21 (8.10)**	**6.51 (7.42)**	**7.23 (8.12)**	**0.093**	**6.95 (7.66)**	**6.51 (7.49)**	**7.39 (7.81)**	**0.116**
Hospital length of stay	**14.95 (17.42)**	**10.47 (12.03)**	**15.13 (17.59)**	**0.31**	**13.96 (31.09)**	**10.63 (12.22)**	**17.28 (42.02)**	**0.215**

Values are presented as mean (standard deviation) for continuous variables and number (percentage) for categorical variables. Variables in bold have *p* < 0.05

**Table 3:** Baseline characteristics before and after propensity score matching of cohort 3

	Before matching				After matching			
	Overall (*N* = 35,188)	36 ℃–38 ℃ (*N* = 27,538)	> 38 ℃ (*N* = 7650)	SMD	Overall (*N* = 14,926)	36 ℃–38 ℃ (*N* = 7463)	> 38 ℃ (*N* = 7463)	SMD
Age	**66.04 (15.92)**	**67.36 (15.37)**	**61.30 (16.94)**	**0.374**	61.79 (16.77)	61.84 (16.82)	61.74 (16.73)	0.006
Gender (Female)	**14,846 (42.19%)**	**11,922 (43.29%)**	**2924 (38.22%)**	**0.103**	5712 (38.27%)	2840 (38.05%)	2872 (38.48%)	0.009
Weight	**82.16 (24.15)**	**81.53 (24.05)**	**84.44 (24.37)**	**0.12**	**84.16 (25.06)**	**84.06 (25.96)**	**84.27 (24.12)**	**0.008**
SOFA score	**6.01 (3.47)**	**5.93 (3.45)**	**6.27 (3.54)**	**0.096**	6.24 (3.58)	6.27 (3.66)	6.21 (3.50)	0.018
Charlson score	**5.50 (3.01)**	**5.71 (2.95)**	**4.72 (3.06)**	**0.331**	4.79 (3.01)	4.80 (2.96)	4.78 (3.05)	0.009
Interventions (boolean for 1st 24 h)								
Mechanical ventilation use (YES)	**17,877 (50.80%)**	**12,990 (47.17%)**	**4887 (63.88%)**	**0.341**	9441 (63.25%)	4731 (63.39%)	4710 (63.11%)	0.006
Vasopressor use (YES)	**14,750 (41.92%)**	**11,293 (41.01%)**	**3457 (45.19%)**	**0.084**	6703 (44.91%)	3364 (45.08%)	3339 (44.74%)	0.007
Sedative use (YES)	**17,476 (49.66%)**	**12,836 (46.61%)**	**4640 (60.65%)**	**0.284**	8943 (59.92%)	4474 (59.95%)	4469 (59.88%)	0.001
Albumin use (YES)	**4980 (14.15%)**	**4144 (15.05%)**	**836 (10.93%)**	**0.123**	1652 (11.07%)	821 (11.00%)	831 (11.13%)	0.004
Comorbidities (boolean)								
HF (YES)	**11,305 (32.13%)**	**9356 (33.97%)**	**1949 (25.48%)**	**0.187**	3928 (26.32%)	2004 (26.85%)	1924 (25.78%)	0.024
AFIB (YES)	**5627 (15.99%)**	**4568 (16.59%)**	**1059 (13.84%)**	**0.076**	2113 (14.16%)	1064 (14.26%)	1049 (14.06%)	0.006
Renal (YES)	**9246 (26.28%)**	**7730 (28.07%)**	**1516 (19.82%)**	**0.194**	3027 (20.28%)	1524 (20.42%)	1503 (20.14%)	0.007
Liver (YES)	**3269 (9.29%)**	**2776 (10.08%)**	**493 (6.44%)**	**0.132**	963 (6.45%)	474 (6.35%)	489 (6.55%)	0.008
COPD (YES)	**5627 (15.99%)**	**4705 (17.09%)**	**922 (12.05%)**	**0.143**	1848 (12.38%)	932 (12.49%)	916 (12.27%)	0.007
CAD (YES)	**10,783 (30.64%)**	**8981 (32.61%)**	**1802 (23.56%)**	**0.203**	3608 (24.17%)	1814 (24.31%)	1794 (24.04%)	0.006
Stroke (YES)	**3912 (11.12%)**	**2853 (10.36%)**	**1059 (13.84%)**	**0.107**	2112 (14.15%)	1077 (14.43%)	1035 (13.87%)	0.016
Malignancy (YES)	**5758 (16.36%)**	**4566 (16.58%)**	**1192 (15.58%)**	**0.027**	2327 (15.59%)	1161 (15.56%)	1166 (15.62%)	0.002
Vital signs (1st 24 h)								
MAP	**82.28 (19.20)**	**82.05 (19.10)**	**83.09 (19.51)**	**0.054**	83.12 (19.30)	83.14 (19.10)	83.11 (19.50)	0.001
Heart rate	**91.67 (20.88)**	**89.78 (20.22)**	**98.50 (21.74)**	**0.416**	97.69 (21.61)	97.64 (21.99)	97.73 (21.22)	0.004
Laboratory tests (1st 24 h)								
WBC	**13.28 (10.88)**	**13.20 (10.44)**	**13.58 (12.35)**	**0.033**	**13.60 (11.35)**	**13.62 (10.23)**	**13.58 (12.37)**	**0.004**
Hemoglobin	**10.26 (2.24)**	**10.18 (2.23)**	**10.52 (2.29)**	**0.152**	10.51 (2.30)	10.51 (2.33)	10.51 (2.27)	0.001
Platelet	**204.38 (116.40)**	**203.46 (115.74)**	**207.71 (118.68)**	**0.036**	207.87 (118.15)	207.80 (117.96)	207.94 (118.35)	0.001
Sodium	**137.81 (7.52)**	**137.69 (7.97)**	**138.24 (5.60)**	**0.08**	138.22 (9.23)	138.24 (11.82)	138.20 (5.55)	0.003
Potassium	**4.27 (0.83)**	**4.30 (0.84)**	**4.17 (0.78)**	**0.169**	4.17 (0.77)	4.17 (0.77)	4.17 (0.78)	0.002
Bicarbonate	**22.62 (5.14)**	**22.65 (5.25)**	**22.50 (4.75)**	**0.03**	22.52 (4.94)	22.50 (5.12)	22.54 (4.75)	0.009
Chloride	**103.52 (7.26)**	**103.33 (7.17)**	**104.19 (7.51)**	**0.117**	104.06 (6.80)	104.01 (6.94)	104.10 (6.65)	0.013
BUN	**30.91 (24.96)**	**32.24 (25.95)**	**26.14 (20.33)**	**0.262**	26.43 (20.81)	26.56 (21.17)	26.30 (20.45)	0.012
Lactate	2.26 (1.83)	2.26 (1.84)	2.27 (1.80)	0.009	2.28 (1.83)	2.29 (1.87)	2.26 (1.79)	0.015
Creatinine	**1.66 (1.71)**	**1.70 (1.76)**	**1.49 (1.51)**	**0.129**	**1.50 (1.57)**	**1.51 (1.63)**	**1.49 (1.52)**	**0.008**
pH	7.36 (0.10)	7.36 (0.10)	7.36 (0.10)	0.011	7.36 (0.10)	7.36 (0.10)	7.36 (0.10)	0.006
PO2	162.59 (115.37)	163.57 (117.01)	159.07 (109.17)	0.04	159.70 (110.40)	159.64 (111.48)	159.75 (109.32)	0.001
PCO2	**42.82 (13.37)**	**42.97 (13.47)**	**42.26 (12.97)**	**0.054**	42.23 (12.93)	42.18 (12.88)	42.29 (12.99)	0.008
Outcomes (boolean)								
28-day mortality (Death)	**7320 (20.80%)**	**5845 (21.23%)**	**1475 (19.28%)**	**0.048**	2929 (19.62%)	1487 (19.92%)	1442 (19.32%)	0.015
ICU mortality (Death)	**4172 (11.86%)**	**3186 (11.57%)**	**986 (12.89%)**	**0.04**	1877 (12.58%)	924 (12.38%)	953 (12.77%)	0.012
In-hospital mortality (Death)	6109 (17.36%)	4773 (17.33%)	1336 (17.46%)	0.003	2592 (17.37%)	1295 (17.35%)	1297 (17.38%)	0.001
Length of stay (days)								
ICU length of stay	**6.06 (7.51)**	**5.73 (7.30)**	**7.23 (8.12)**	**0.195**	**6.96 (8.33)**	**6.74 (8.57)**	**7.17 (8.07)**	**0.051**
Hospital length of stay	**13.27 (14.97)**	**12.75 (14.12)**	**15.13 (17.59)**	**0.149**	**14.55 (16.37)**	**14.08 (15.51)**	**15.01 (17.18)**	**0.057**

Values are presented as mean (standard deviation) for continuous variables and number (percentage) for categorical variables. Variables in bold have *p* < 0.05

**Fig. 2:**
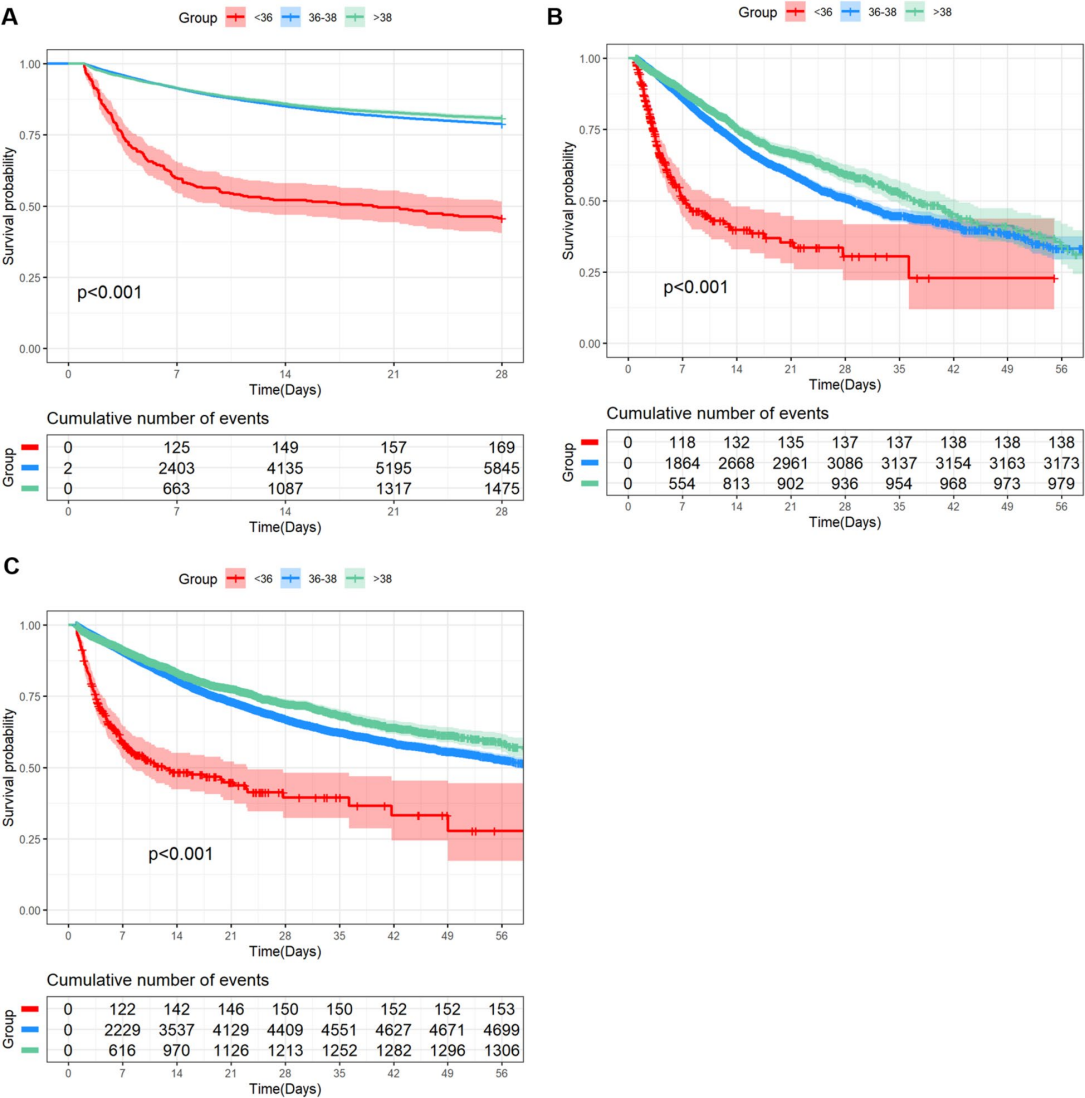
Kaplan–Meier survival curves showing differences in clinical outcomes among patients with sepsis. Kaplan–Meier survival curves for the 28-day (**A**), ICU (**B**), and in-hospital (**C**) mortality among patients with sepsis

Patients with a body temperature of less than 36 °C had a higher SOFA score (< 36 °C vs 36 °C–38 °C, 8.58 ± 3.88 vs 5.93 ± 3.45) (< 36 °C vs > 38 °C, 8.58 ± 3.88 vs 6.27 ± 3.54) compared with the other two groups, and a higher proportion of patients received mechanical ventilation (< 36 °C vs 36 °C–38 °C, 74.60% vs 47.17%)(< 36 °C vs > 38 °C, 74.60% vs 63.88%), vasopressors (< 36 °C vs 36 °C–38 °C, 64.31% vs 41.01%)(< 36 °C vs > 38° C, 64.31% vs 45.19%), sedatives (< 36 °C vs 36 °C–38 °C, 66.56% vs 46.61%)(< 36 °C vs > 38 °C, 6.56% vs 60.65%), and albumin(< 36 °C vs 36 °C–38 °C, 16.08% vs 15.05%)(< 36 °C vs > 38 °C, 16.08% vs 10.93%) within 24 h of ICU admission. Furthermore, laboratory tests indicated that patients with hypothermia exhibited higher levels of WBC (< 36 °C vs 36 °C–38 °C, 14.83 K/μL vs 13.20 K/μL) (< 36 °C vs > 38 °C, 14.83 K/μL vs 13.58 K/μL), BUN (< 36 °C vs 36 °C–38 °C, 38.40 mg/dL vs 32.24 mg/dL) (< 36 °C vs > 38 °C, 38.40 mg/dL vs 26.14 mg/dL), lactate (< 36 °C vs 36 °C–38 °C, 4.08 mmol/L vs 2.26 mmol/L) (< 36 °C vs > 38 °C, 4.08 mmol/L vs 2.27 mmol/L), and creatinine(< 36 °C vs 36 °C–38 °C, 2.20 mg/dL vs 1.70 mg/dL) (< 36 °C vs > 38 °C, 2.20 mg/dL vs 1.49 mg/dL) in their initial measurements within 24 h of ICU admission. Kaplan–Meier curves indicated a significantly worse prognosis for hypothermic patients (*p* < 0.001) (Fig. 2) (Table S2-4).

Patients with a temperature exceeding 38 °C were more likely to require mechanical ventilation (63.88% vs 47.17%), vasopressors (45.19% vs 41.01%), and sedatives (63.88% vs 47.17%), and had a higher likelihood of experiencing a stroke (13.84% vs 10.36%) compared to those with normal body temperature. However, the incidence of HF (25.48% vs 33.97%), AFIB (13.84% vs 16.59%) and CAD (23.56% vs 32.61%) was lower in this group.

**Fig. 3:**
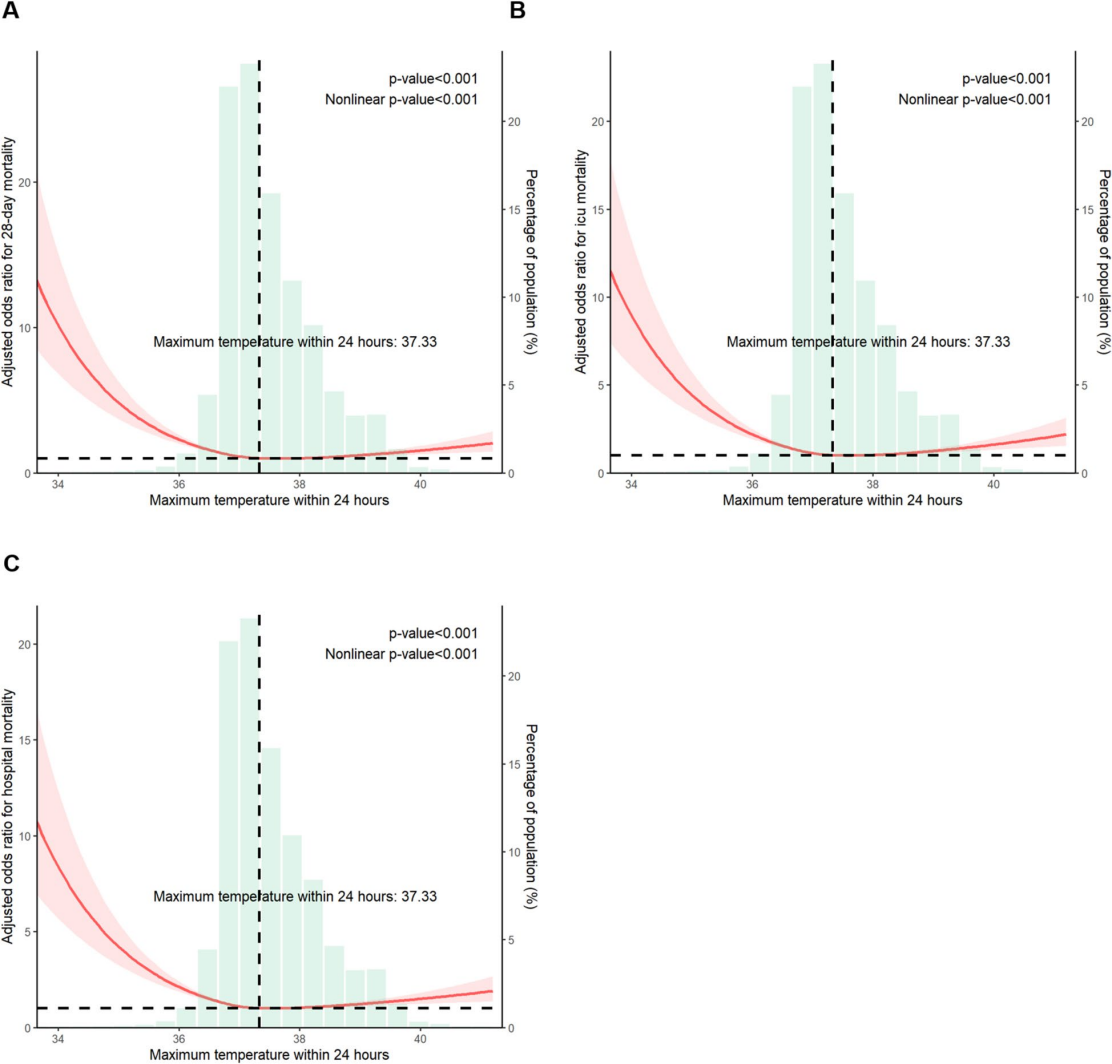
RCS curve for the maximum body temperature and clinical outcome. Solid red lines are odds ratios, with light red regions showing 95% confidence. RCS curve for the body temperature and the 28-day (**A**), ICU (**B**), in-hospital (**C**) mortality

Additionally, the RCS analysis revealed a non-linear, U-shaped relationship between the maximum body temperatures recorded within 24 h of ICU admission and clinical outcomes, including 28-day, ICU, and in-hospital mortality. Patients with sepsis exhibit the best prognosis when their body temperature reaches 37.33 ℃. The odds ratio (OR) increases when the temperature deviates either below or above this value (Fig. 3). Furthermore, the data analysis revealed a comparable non-linear relationship between minimum body temperature and clinical outcomes in sepsis patients within the first 24 h of ICU admission (Figure S2).

### Primary outcome

**Fig. 4:**
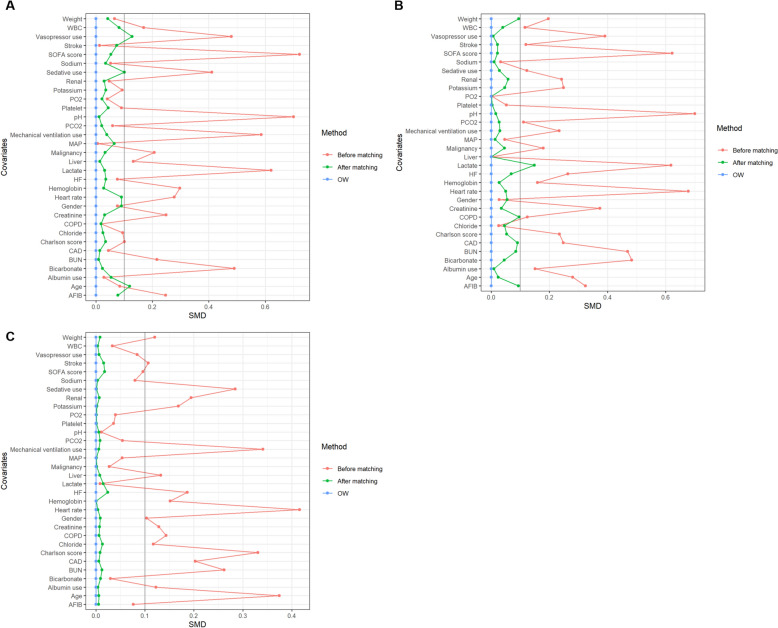
Change in standardized mean difference (SMD) before and after matching. Change in SMD before and after matching of cohort 1 (**A**), cohort 2 (**B**), cohort 3 (**C**)

Following multiple imputation of missing data, we conducted a multivariable logistic regression analysis, revealing that patients with hypothermia exhibited a lower 28-day mortality rate compared to those with normal (OR 0.25, 95%CI 0.19–0.32, *p* < 0.001) or elevated body temperatures (OR 0.26, 95%CI 0.19–0.34, *p* < 0.001). However, no significant difference was observed between individuals with normal body temperature and those with hyperthermia (OR 0.98, 95%CI 0.91–1.05, *p* < 0.714). Utilizing the estimated propensity score, overlap weighting (OW) was applied to standardize differences between cohorts. Post-normalization, the imbalance of covariates among the three groups was notably reduced (Fig. 4, Table S5–7), yet the findings remained consistent (Table 4).

**Table 4:** Primary and secondary outcome analyses with different models for 3 cohorts

	Cohort 1^a^	*p*-value	Cohort 2^b^	*p*-value	Cohort 3^c^	*p*-value
28-day mortality						
Log-rank test [HR (95% CI)]	**0.27 (0.20, 0.36)**	**< 0.001**	**0.25 (0.18, 0.33)**	**< 0.001**	**0.90 (0.86, 0.96)**	**< 0.001**
Multivariate Cox model adjusted with all covariates [HR (95% CI)]	**0.36 (0.31, 0.42)**	**< 0.001**	**0.38 (0.32, 0.46)**	**< 0.001**	1 (0.94, 1.06)	0.963
Cox model adjusted with OW [HR (95% CI)]	**0.37 (0.31, 0.44)**	**< 0.001**	**0.41 (0.34, 0.50)**	**< 0.001**	1 (0.94, 1.06)	0.88
Multivariate logistic model adjusted with all covariates [OR (95% CI)]	**0.25 (0.19, 0.32)**	**< 0.001**	**0.26 (0.19, 0.34)**	**< 0.001**	0.98 (0.91, 1.05)	0.572
Logistic model adjusted with OW [OR (95% CI)]	**0.28 (0.19, 0.41)**	**< 0.001**	**0.29 (0.19, 0.43)**	**< 0.001**	0.96 (0.87, 1.07)	0.487
Doubly robust estimation model [OR (95% CI)]	**0.28 (0.21, 0.36)**	**< 0.001**	**0.29 (0.21, 0.38)**	**< 0.001**	0.96 (0.89, 1.04)	0.327
Propensity score matching [OR (95% CI)]	**0.32 (0.22, 0.48)**	**< 0.001**	**0.35 (0.24, 0.50)**	**< 0.001**	0.96 (0.89, 1.04)	0.359
ICU mortality						
Log-rank test [HR (95% CI)]	**0.30 (0.22, 0.41)**	**< 0.001**	**0.26 (0.19, 0.35)**	**< 0.001**	**0.84 (0.79, 0.90)**	**< 0.001**
Multivariate Cox model adjusted with all covariates [HR (95% CI)]	**0.43 (0.36, 0.5)**	**< 0.001**	**0.42 (0.34, 0.51)**	**< 0.001**	0.98 (0.91, 1.05)	0.564
Cox model adjusted with OW [HR (95% CI)]	**0.4 (0.32, 0.49)**	**< 0.001**	**0.44 (0.35, 0.55)**	**< 0.001**	0.98 (0.91, 1.06)	0.691
Multivariate logistic model adjusted with all covariates [OR (95% CI)]	**0.25 (0.19, 0.32)**	**< 0.001**	**0.27 (0.21, 0.36)**	**< 0.001**	1.07 (0.98, 1.17)	0.124
Logistic model adjusted with OW [OR (95% CI)]	**0.27 (0.17, 0.40)**	**< 0.001**	**0.3 (0.19, 0.47)**	**< 0.001**	1.06 (0.94, 1.20)	0.336
Doubly robust estimation model [OR (95% CI)]	**0.27 (0.20, 0.35)**	**< 0.001**	**0.3 (0.22, 0.41)**	**< 0.001**	1.06 (0.97, 1.16)	0.173
Propensity score matching [OR (95% CI)]	**0.26 (0.17, 0.41)**	**< 0.001**	**0.39 (0.27, 0.57)**	**< 0.001**	1.04 (0.94, 1.14)	0.486
In-hospital mortality						
Log-rank test [HR (95% CI)]	**0.29 (0.21, 0.39)**	**< 0.001**	**0.25 (0.18, 0.34)**	**< 0.001**	**0.86 (0.81, 0.91)**	**< 0.001**
Multivariate Cox model adjusted with all covariates [HR (95% CI)]	**0.4 (0.34, 0.47)**	**< 0.001**	**0.41 (0.34, 0.50)**	**< 0.001**	0.98 (0.92, 1.04)	0.463
Cox model adjusted with OW [HR (95% CI)]	**0.39 (0.32, 0.48)**	**< 0.001**	**0.43 (0.35, 0.54)**	**< 0.001**	0.98 (0.91, 1.04)	0.491
Multivariate logistic model adjusted with all covariates [OR (95% CI)]	**0.28 (0.21, 0.36)**	**< 0.001**	**0.29 (0.22, 0.38)**	**< 0.001**	1.03 (0.95, 1.11)	0.469
Logistic model adjusted with OW [OR (95% CI)]	**0.3 (0.20, 0.44)**	**< 0.001**	**0.31 (0.20, 0.48)**	**< 0.001**	1.02 (0.91, 1.14)	0.734
Doubly robust estimation model [OR (95% CI)]	**0.3 (0.23, 0.39)**	**< 0.001**	**0.31 (0.23, 0.42)**	**< 0.001**	1.02 (0.94, 1.10)	0.632
Propensity score matching [OR (95% CI)]	**0.29 (0.19, 0.45)**	**< 0.001**	**0.42 (0.30, 0.60)**	**< 0.001**	1 (0.92, 1.09)	0.983

Statistical analyses of different models with *p* < 0.05 were displayed in bold

HR: hazard ratio; CI: confidence interval

^a^Cohort 1: < 36 ℃ versus 36–38 ℃

^b^Cohort 2: < 36 ℃ versus > 38 ℃

^c^Cohort 3: 36–38 ℃ versus > 38 ℃

### Secondary outcome and sensitivity analyses

Hypothermia was also linked to reduced in-hospital and ICU mortality. Sensitivity analyses were conducted across all assessment models, including multiple Cox regression, OW-adjusted Cox regression, multiple logistic regression, OW-adjusted multiple logistic regression and doubly robust estimation. McNemar test was performed on the matched data. All analyses reached the same conclusion: hypothermic patients had lower 28-day mortality compared to those with normothermia or hyperthermia. However, across all models, differences in clinical outcomes between normothermic and hyperthermic patients were observed only in the log-rank analysis, while no significant differences were found in other models (Table 4).

### Subgroup analyses

**Fig. 5:**
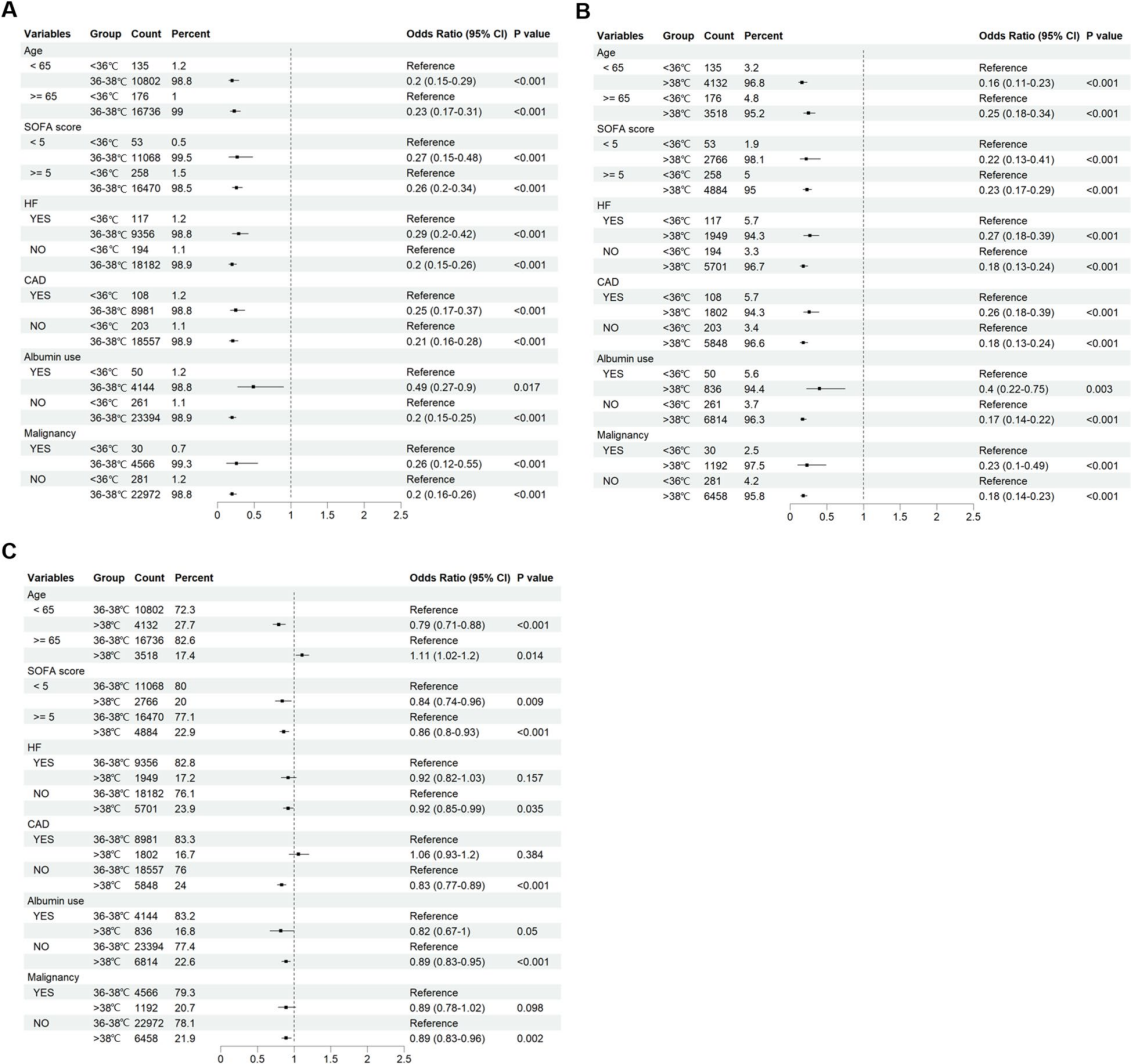
Subgroup analysis of the association between the body temperature and primary outcome. Subgroup analysis of the association between body temperature and 28-day mortality in cohort 1 (**A**), cohort 2 (**B**), cohort 3 (**C**)

Subgroup analysis showed that hypothermia patients had the highest risk of 28-day mortality, ICU mortality, and in-hospital mortality in almost all subgroups. In patients younger than 65 years of age, hyperthermia emerged as a protective factor against 28-day mortality relative to normal body temperature (OR 0.79, 95%CI 0.71–0.88, *p* < 0.001), whereas the opposite was true in older patients older than or equal to 65 years of age (OR 1.11, 95%CI 1.02–1.2, *p* = 0.014). This phenomenon is also generally consistent in the analysis of ICU mortality and in-hospital mortality. In coronary artery disease patients, hyperthermia was associated with higher ICU (OR 1.42, 95%CI 1.23–1.64, *p* < 0.001) and in-hospital mortality (OR 1.29, 95%CI 1.13–1.46, *p* < 0.001), but had no significant effect on 28-day mortality (OR 1.06, 95%CI 0.93–1.2, *p* = 0.384). Regardless of albumin use, lower body temperature was a risk factor for clinical outcomes in sepsis patients when compared to those with normal or elevated body temperatures. In patients with non-malignant tumors, hyperthermia emerged as a protective factor against 28-day mortality (OR 0.89, 95%CI 0.83–0.96, *p* = 0.002) compared with patients with normal body temperature, but this phenomenon was not evident in patients with malignant tumors (OR 0.89, 95%CI 0.78–1.02, *p* = 0.098) (Fig. 5) (Figure S1).

## Discussion

This study suggests a non-linear relationship between the maximum body temperature within the first 24 h of admission and clinical outcomes in patients with sepsis. Patients with hypothermia had a worse prognosis compared to those with normal or elevated temperatures. In non-elderly patients (< 65 years old) with sepsis, hyperthermia appeared to be a protective factor, while in elderly patients (≥ 65 years old), the opposite trend was observed, though without significant differences. These results may be linked to altered immune responses in sepsis patients.

Hypothermia is strongly associated with poor outcomes in sepsis, consistent with previous findings. Numerous clinical studies have shown that sepsis patients with hypothermia experience higher mortality rates and longer hospital stays than those with normothermia, likely due to weakened immune responses, organ dysfunction, and diminished infection control [12, 13]. A multicenter prospective study by Shigeki Kushimoto et al. demonstrated that differences in 28-day mortality were evident between hypothermic and non-hypothermic patients [14]. Additionally, a retrospective analysis of 525 sepsis patients in Dutch ICUs identified hypothermia within the first 24 h as a risk factor for outcomes including 30-day, 60-day and 1-year mortality, as well as acute kidney injury (AKI) incidence. The poor prognosis associated with hypothermia may be linked to persistent lymphocytopenia [15], elevated markers of endothelial activation [16], and hypertension. In our study, we also observed that the hypothermia group had a higher proportion of white blood cells, as well as an increased risk of stroke and coronary artery disease.

Furthermore, prior basic studies have suggested that the onset of hypothermia-type sepsis may be associated with vasodilatory hypothermia and a lethally hypodynamic state induced by nitric oxide synthase (iNOS)-derived nitric oxide (NO) in late sepsis [17]. Additionally, research has examined the inhibitory effects on the immune system, particularly the decline in neutrophil function and phagocytosis, which may restrict the body's capacity to effectively eliminate sources of infection. During infection, polymorphonuclear cells (PMNs), such as neutrophils, form neutrophil extracellular traps (NETs) to contain and eliminate pathogens. Studies have demonstrated that NET formation is impaired at 35 °C and 42 °C, while optimal at 40 °C, highlighting how temperature affects immune response [18]. This evidence underscores the critical importance of managing hypothermia in sepsis treatment. Additionally, hypothermia is uncommon at the time of death, even when multi-organ failure is at its most severe [19, 20]. Therefore, the association between hypothermia and higher mortality rates may not necessarily indicate that hypothermia exacerbates the dysfunction caused by sepsis. This hypothesis requires further investigation and discussion for confirmation.

Contrary to the established view of hypothermia as a poor prognostic factor, the role of hyperthermia in sepsis remains debated. On one hand, moderate increases in body temperature can inhibit bacterial growth, enhance immune function by promoting antibody and cytokine synthesis, and help eliminate pathogens, providing protective effects. A clinical study that combined data from two large, independent multinational ICU cohorts (636051 patients) found that elevated body temperature was associated with reduced in-hospital mortality, with the lowest risk of death observed in patients whose peak temperature ranged from 39.0 °C to 39.4 °C [21]. On the other hand, persistent high fever may exacerbate the inflammatory response, precipitating a cytokine storm, disrupting the body's immune regulation, and elevating metabolic load, ultimately resulting in organ damage and multiple organ dysfunction. Laupland et al. found that patients with high temperatures (> 39.5 °C) were more likely to experience arrhythmias and tachycardia, resulting in worse clinical outcomes [4]. Similarly, a large epidemiological study linked high fever (≥ 39.5 °C) with significantly increased mortality in sepsis patients [22]. Moderate temperature regulation, such as mild hypothermia, can enhance blood coagulation in patients with sepsis [23] and diminish the extent of systemic organ failure [24].

The paradoxical outcome may be driven by various factors, including the patient's age, the presence of specific complications (such as malignancy, stroke) [25], outside temperature [26], and the patient's gut microbiota composition [27]. A multicenter retrospective study indicated that hyperthermia (BT ≥ 38.3 °C) was a protective factor for 28-day mortality in sepsis patients under 75 years old, while in older patients, fever had no significant impact on clinical outcomes, possibly due to reduced thermogenic cytokine production and frequent comorbidities that impair thermoregulation [28]. Comparable results were observed in my study. Moreover, fever is a common symptom in patients with hematological malignancies and some solid tumors, such as primary liver cancer, renal cell carcinoma, and sarcoma, potentially related to cancer-induced alterations in cytokine activity [29]. Sepsis patients with malignancies typically have a poorer prognosis. Subgroup analyses in this study revealed that hyperthermia was a protective factor for 28-day mortality in patients without malignancy, but not significantly so in those with malignancy, consistent with findings from previous research.

Given the heterogeneity of body temperature fluctuations during sepsis, managing temperature effectively poses a significant challenge for clinicians. For patients with elevated temperatures, it is generally accepted that appropriate cooling measures are essential to protect vital organs, such as the brain and heart, which are particularly sensitive to temperature increases. Studies have demonstrated that external cooling can reduce vasopressor requirements in septic shock patients and lower 14-day mortality [30]. However, the necessity of antipyretic treatment in febrile sepsis patients remains contentious. A multicenter randomized clinical trial involving 700 patients with infections and temperatures exceeding 38.3 °C found that early intravenous acetaminophen had no significant impact on 90-day mortality or ICU-free days [31]. Furthermore, while rewarming therapy is widely considered beneficial, there remains a lack of robust clinical and experimental evidence to elucidate its role and mechanisms in sepsis treatment [32]. Induced hypothermia therapy has also drawn considerable interest in recent research. Despite promising outcomes in animal models [33–35], randomized clinical trials have shown that lowering core body temperature to 32–34 °C did not improve mortality [36]. As such, further research is necessary to inform clinical decisions regarding temperature management in sepsis patients, and this study offers a certain degree of clinical foundation for such considerations.

However, this study has certain limitations. First, the RCS analysis showed a non-linear relationship between peak body temperature within the first 24 h of admission and clinical outcome, with optimal prognosis observed at 37.33 °C. However, a single temperature measurement may not fully capture the patient’s condition. For example, trauma patients exhibit complex circadian rhythms in temperature, and early increases in rhythm frequency and amplitude are associated with sepsis development [37]. Additionally, Bhavani and colleagues employed longitudinal temperature trajectories to categorize hospitalized patients with infections into four distinct subtypes: “hyperthermic, slow resolvers”, “hyperthermic, fast resolvers”, “normothermic”, and “hypothermic”. Their analysis revealed significant differences in inflammatory markers (such as C-reactive protein and erythrocyte sedimentation rate) and clinical outcomes across these subtypes [9]. Thus, real-time temperature monitoring may be necessary to better understand the immunophenotype of infected patients [9, 38]. Second, previous studies have indicated that sepsis complications can affect temperature fluctuations. Subgroup analyses of coronary heart disease, atrial fibrillation, and malignancy were conducted. The findings indicated no significant differences in mortality. Further investigation is required to examine the effects of body temperature variations on clinical outcomes in more specific subgroups, such as those with stroke, solid tumors, and hematologic malignancies.

## Conclusions

In summary, the results demonstrated that hypothermia was linked to a higher risk-adjusted 28-day, ICU, and in-hospital mortality in sepsis patients. Among non-elderly patients, elevated body temperature served as a protective factor against sepsis compared to normothermia, whereas in elderly patients, the opposite trend was observed, though without statistical significance. This study elucidated the relationship between body temperature fluctuations and clinical outcomes, offering valuable insights and recommendations for temperature management in sepsis.

## Supplementary Information

Supplementary Material 1: Figure S1 Subgroup analysis of the association between the body temperature and secondary outcome. A, B Subgroup analysis of the association between body temperature and ICU, in-hospital mortalityin cohort 1. C, D Subgroup analysis of the association between body temperature and ICU, in-hospital mortalityin cohort 2. E, F Subgroup analysis of the association between body temperature and ICU, in-hospital mortalityin cohort 3

Supplementary Material 2: Figure S2 RCS curve for the minimum body temperature and clinical outcome. Solid red lines are odds ratios, with light red regions showing 95% confidence. RCS curve for the body temperature and the 28-day, ICU, in-hospitalmortality

Supplementary Material 3

Supplementary Material 4

## Data Availability

The datasets presented in the current study are available in the MIMIC-IV database (https://physionet.org/content/mimiciv/3.0/).

## References

[1] Machado FR, Cavalcanti AB, Bozza FA, Ferreira EM, Angotti Carrara FS, Sousa JL, et al. The epidemiology of sepsis in Brazilian intensive care units (the Sepsis PREvalence Assessment Database, SPREAD): an observational study. Lancet Infect Dis. 2017;17(11):1180–9. 28826588 10.1016/S1473-3099(17)30322-5

[2] Hotchkiss RS, Moldawer LL, Opal SM, Reinhart K, Turnbull IR, Vincent JL. Sepsis and septic shock. Nat Rev Dis Prim. 2016;2:16045. 28117397 10.1038/nrdp.2016.45 PMC5538252

[3] Liu D, Huang SY, Sun JH, Zhang HC, Cai QL, Gao C, et al. Sepsis-induced immunosuppression: mechanisms, diagnosis and current treatment options. Mil Med Res. 2022;9(1):56. 36209190 10.1186/s40779-022-00422-y PMC9547753

[4] Laupland KB, Shahpori R, Kirkpatrick AW, Ross T, Gregson DB, Stelfox HT. Occurrence and outcome of fever in critically ill adults. Crit Care Med. 2008;36(5):1531–5. 18434882 10.1097/CCM.0b013e318170efd3

[5] Niven DJ, Laupland KB. Pyrexia: aetiology in the ICU. Crit Care. 2016;20(1):247. 27581757 10.1186/s13054-016-1406-2 PMC5007859

[6] Annane D. Body temperature in sepsis: a hot topic. Lancet Respir Med. 2018;6(3):162–3. 29325752 10.1016/S2213-2600(18)30003-1

[7] Leijte GP, Kox M, Pickkers P. Fever in sepsis: still a hot topic. Am J Respir Crit Care Med. 2019;200(2):263–4. 30908926 10.1164/rccm.201903-0484LE PMC6635785

[8] Walter EJ, Hanna-Jumma S, Carraretto M, Forni L. The pathophysiological basis and consequences of fever. Crit Care. 2016;20(1):200. 27411542 10.1186/s13054-016-1375-5 PMC4944485

[9] Bhavani SV, Carey KA, Gilbert ER, Afshar M, Verhoef PA, Churpek MM. Identifying novel sepsis subphenotypes using temperature trajectories. Am J Respir Crit Care Med. 2019;200(3):327–35. 30789749 10.1164/rccm.201806-1197OC PMC6680307

[10] Gopalakrishnan C, Bykov K, Fischer MA, Connolly JG, Gagne JJ, Fralick M. Association of fluoroquinolones with the risk of aortic aneurysm or aortic dissection. JAMA Intern Med. 2020;180(12):1596–605. 32897307 10.1001/jamainternmed.2020.4199 PMC7489402

[11] Patorno E, Goldfine AB, Schneeweiss S, Everett BM, Glynn RJ, Liu J, et al. Cardiovascular outcomes associated with canagliflozin versus other non-gliflozin antidiabetic drugs: population based cohort study. BMJ. 2018;360: k119. 29437648 10.1136/bmj.k119 PMC5799855

[12] Ramgopal S, Horvat CM, Adler MD. Association of triage hypothermia with in-hospital mortality among patients in the emergency department with suspected sepsis. J Crit Care. 2020;60:27–31. 32731103 10.1016/j.jcrc.2020.07.011 PMC7872398

[13] Clemmer TP, Fisher CJ Jr, Bone RC, Slotman GJ, Metz CA, Thomas FO. Hypothermia in the sepsis syndrome and clinical outcome. The Methylprednisolone Severe Sepsis Study Group. Crit Care Med. 1992;20(10):1395–401. 1395659 10.1097/00003246-199210000-00006

[14] Kushimoto S, Gando S, Saitoh D, Mayumi T, Ogura H, Fujishima S, et al. The impact of body temperature abnormalities on the disease severity and outcome in patients with severe sepsis: an analysis from a multicenter, prospective survey of severe sepsis. Crit Care. 2013;17(6):R271. 24220071 10.1186/cc13106 PMC4057086

[15] Drewry AM, Fuller BM, Skrupky LP, Hotchkiss RS. The presence of hypothermia within 24 hours of sepsis diagnosis predicts persistent lymphopenia. Crit Care Med. 2015;43(6):1165–9. 25793436 10.1097/CCM.0000000000000940 PMC4700928

[16] Wiewel MA, Harmon MB, van Vught LA, Scicluna BP, Hoogendijk AJ, Horn J, et al. Risk factors, host response and outcome of hypothermic sepsis. Crit Care. 2016;20(1):328. 27737683 10.1186/s13054-016-1510-3 PMC5064908

[17] Takatani Y, Ono K, Suzuki H, Inaba M, Sawada M, Matsuda N. Inducible nitric oxide synthase during the late phase of sepsis is associated with hypothermia and immune cell migration. Lab Invest. 2018;98(5):629–39. 29449632 10.1038/s41374-018-0021-z

[18] Janko J, Bečka E, Kmeťová K, Hudecová L, Konečná B, Celec P, et al. Neutrophil extracellular traps formation and clearance is enhanced in fever and attenuated in hypothermia. Front Immunol. 2023;14:1257422. 37849757 10.3389/fimmu.2023.1257422 PMC10577177

[19] Romanovsky AA, Székely M. Fever and hypothermia: two adaptive thermoregulatory responses to systemic inflammation. Med Hypotheses. 1998;50(3):219–26. 9578327 10.1016/s0306-9877(98)90022-6

[20] Steiner AA, Fonseca MT, Soriano FG. Should we assume that hypothermia is a dysfunction in sepsis? Crit Care. 2017;21(1):8. 28073371 10.1186/s13054-016-1584-y PMC5225576

[21] Young PJ, Saxena M, Beasley R, Bellomo R, Bailey M, Pilcher D, et al. Early peak temperature and mortality in critically ill patients with or without infection. Intensive Care Med. 2012. 10.1007/s00134-012-2478-3. 22290072 10.1007/s00134-012-2478-3

[22] Lee BH, Inui D, Suh GY, Kim JY, Kwon JY, Park J, et al. Association of body temperature and antipyretic treatments with mortality of critically ill patients with and without sepsis: multi-centered prospective observational study. Crit Care. 2012;16(1):R33. 22373120 10.1186/cc11211 PMC3396278

[23] Johansen ME, Jensen JU, Bestle MH, Ostrowski SR, Thormar K, Christensen H, et al. Mild induced hypothermia: effects on sepsis-related coagulopathy—results from a randomized controlled trial. Thromb Res. 2015;135(1):175–82. 25466837 10.1016/j.thromres.2014.10.028

[24] Jiang L, Li X, Hu J, Tang Z. Mild hypothermia alleviates CLP-induced multiple organ dysfunction by mitigating pyroptosis through the TLR4/NF-κB/NLRP3 signaling pathway. Arch Med Res. 2023;54(1):7–16. 36588003 10.1016/j.arcmed.2022.11.005

[25] Cheshire WP Jr. Thermoregulatory disorders and illness related to heat and cold stress. Auton Neurosci. 2016;196:91–104. 26794588 10.1016/j.autneu.2016.01.001

[26] Benzoni NS, Carey KA, Bewley AF, Klaus J, Fuller BM, Edelson DP, et al. Temperature trajectory subphenotypes in oncology patients with neutropenia and suspected infection. Am J Respir Crit Care Med. 2023;207(10):1300–9. 36449534 10.1164/rccm.202205-0920OC PMC10595453

[27] Bongers KS, Chanderraj R, Woods RJ, McDonald RA, Adame MD, Falkowski NR, et al. The gut microbiome modulates body temperature both in sepsis and health. Am J Respir Crit Care Med. 2023;207(8):1030–41. 36378114 10.1164/rccm.202201-0161OC PMC10112447

[28] Shimazui T, Nakada TA, Walley KR, Oshima T, Abe T, Ogura H, et al. Significance of body temperature in elderly patients with sepsis. Crit Care. 2020;24(1):387. 32605659 10.1186/s13054-020-02976-6 PMC7329464

[29] Sørensen HT, Mellemkjaer L, Skriver MV, Johnsen SP, Nørgård B, Olsen JH, et al. Fever of unknown origin and cancer: a population-based study. Lancet Oncol. 2005;6(11):851–5. 16257792 10.1016/S1470-2045(05)70346-6

[30] Schortgen F, Clabault K, Katsahian S, Devaquet J, Mercat A, Deye N, et al. Fever control using external cooling in septic shock: a randomized controlled trial. Am J Respir Crit Care Med. 2012;185(10):1088–95. 22366046 10.1164/rccm.201110-1820OC

[31] Young P, Saxena M, Bellomo R, Freebairn R, Hammond N, van Haren F, et al. Acetaminophen for fever in critically ill patients with suspected infection. N Engl J Med. 2015;373(23):2215–24. 26436473 10.1056/NEJMoa1508375

[32] Harmon MBA, Pelleboer I, Steiner AA, Wiewel M, Schultz MJ, Horn J, et al. Opinions and management of hypothermic sepsis: results from an online survey. Ther Hypothermia Temp Manag. 2020;10(2):102–5. 31233381 10.1089/ther.2019.0002

[33] L’Her E, Amerand A, Vettier A, Sebert P. Effects of mild induced hypothermia during experimental sepsis. Crit Care Med. 2006;34(10):2621–3. 16915110 10.1097/01.CCM.0000240231.76837.DC

[34] Léon K, Moisan C, Amérand A, Poupon G, L’Her E. Effect of induced mild hypothermia on two pro-inflammatory cytokines and oxidative parameters during experimental acute sepsis. Redox Rep. 2013;18(3):120–6. 23746123 10.1179/1351000213Y.0000000049 PMC6837679

[35] Ding W, Shen Y, Li Q, Jiang S, Shen H. Therapeutic mild hypothermia improves early outcomes in rats subjected to severe sepsis. Life Sci. 2018;199:1–9. 29505782 10.1016/j.lfs.2018.03.002

[36] Itenov TS, Johansen ME, Bestle M, Thormar K, Hein L, Gyldensted L, et al. Induced hypothermia in patients with septic shock and respiratory failure (CASS): a randomised, controlled, open-label trial. Lancet Respir Med. 2018;6(3):183–92. 29325753 10.1016/S2213-2600(18)30004-3 PMC10928558

[37] Coiffard B, Diallo AB, Culver A, Antonini F, Hammad E, Leone M, et al. Exacerbation of circadian rhythms of core body temperature and sepsis in trauma patients. J Crit Care. 2020;60:23–6. 32731102 10.1016/j.jcrc.2020.07.010

[38] Fonseca MT, Rodrigues AC, Cezar LC, Fujita A, Soriano FG, Steiner AA. Spontaneous hypothermia in human sepsis is a transient, self-limiting, and nonterminal response. J Appl Physiol. 2016;120(12):1394–401. 26989218 10.1152/japplphysiol.00004.2016

