# Supplementary figures and images for: Association between body temperature and all-cause mortality in patients with sepsis: analysis of the MIMIC-IV database
Source: Eur J Med Res. 2024 Dec 26;29:630. doi: 10.1186/s40001-024-02219-2 (PMC11673708; doi:10.1186/s40001-024-02219-2)

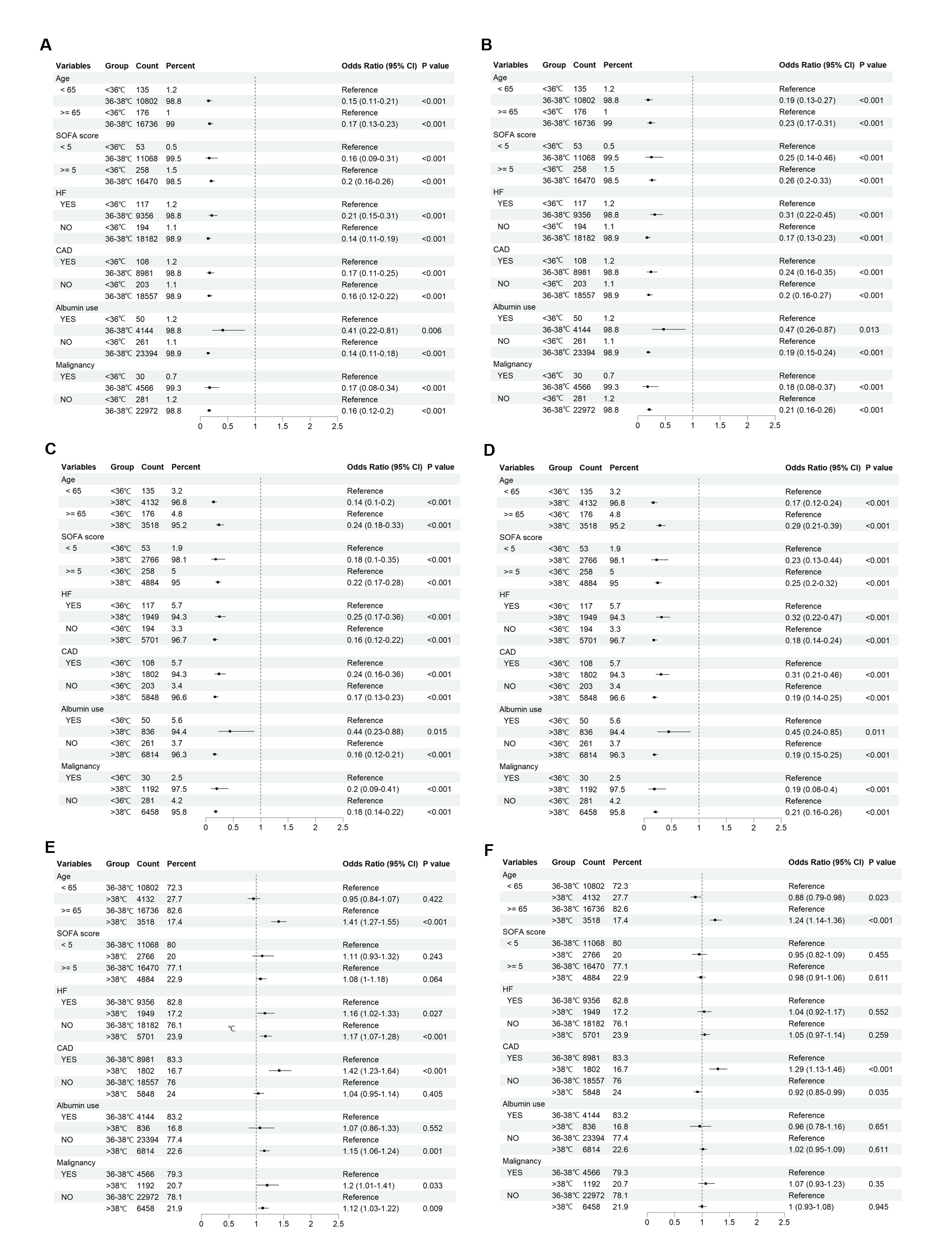

Supplement: Supplementary file 1 — Supplementary Material 1: Figure S1 Subgroup analysis of the association between the body temperature and secondary outcome. A, B Subgroup analysis of the association between body temperature and ICU, in-hospital mortalityin cohort 1. C, D Subgroup analysis of the association between body temperature and ICU, in-hospital mortalityin cohort 2. E, F Subgroup analysis of the association between body temperature and ICU, in-hospital mortalityin cohort 3 [file 40001_2024_2219_MOESM1_ESM.tif]

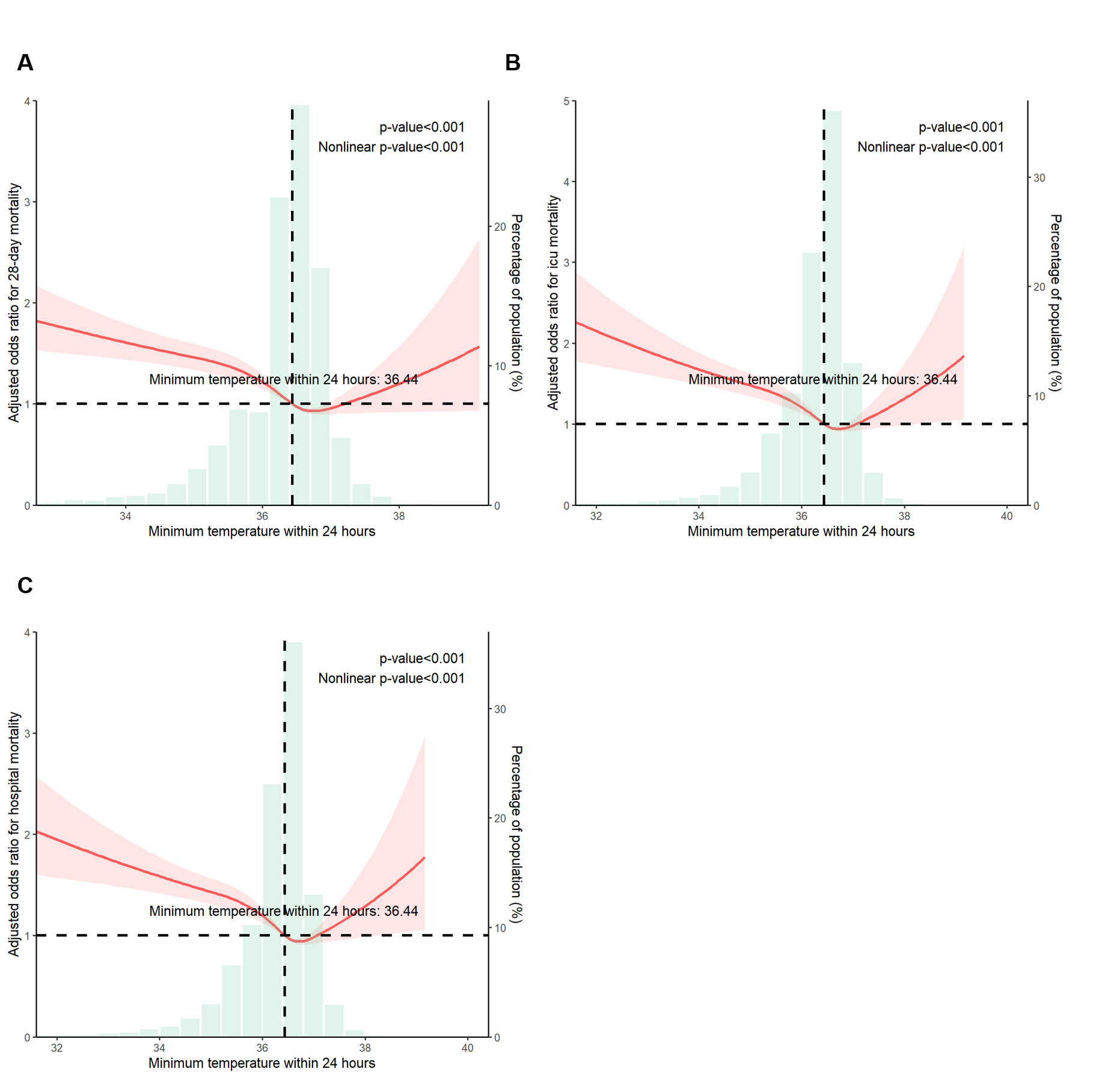

Supplement: Supplementary file 2 — Supplementary Material 2: Figure S2 RCS curve for the minimum body temperature and clinical outcome. Solid red lines are odds ratios, with light red regions showing 95% confidence. RCS curve for the body temperature and the 28-day, ICU, in-hospitalmortality [file 40001_2024_2219_MOESM2_ESM.tif]
