# Supplementary material for: Association between body temperature and all-cause mortality in patients with sepsis: analysis of the MIMIC-IV database
Source: Eur J Med Res. 2024 Dec 26;29:630. doi: 10.1186/s40001-024-02219-2 (PMC11673708; doi:10.1186/s40001-024-02219-2)
Supplement: Supplementary file 4 — Supplementary Material 4 [file 40001_2024_2219_MOESM4_ESM.docx]

Supplementary Table 1. Basic demographic characteristics of the original cohort

|  | **<36℃ (N=311)** | **36-38℃ (N=27538)** | **>38℃ (N=7650)** | **p-value** | **SMD (Compare to <36℃, respectively)** | **Missing data (%)** |
| --- | --- | --- | --- | --- | --- | --- |
| Age | **68.00 [55.00, 79.50]** | **69.00 [58.00, 79.00]** | **63.00 [51.00, 74.00]** | **<0.001** | **[0.085, 0.281]** | **0.00** |
| Gender (Female) | **123 (39.55%)** | **11922 (43.29%)** | **2924 (38.22%)** | **<0.001** | **[0.076, 0.027]** | **0.00** |
| Weight | **78.65 [66.91, 90.50]** | **78.08 [65.30, 93.71]** | **80.42 [68.52, 96.93]** | **<0.001** | **[0.067, 0.197]** | **0.26** |
| SOFA score | **8.00 [5.50, 11.00]** | **5.00 [3.00, 8.00]** | **6.00 [4.00, 8.00]** | **<0.001** | **[0.722, 0.623]** | **0.00** |
| Charlson score | **5.00 [3.00, 7.50]** | **6.00 [4.00, 8.00]** | **4.00 [2.00, 7.00]** | **<0.001** | **[0.101, 0.234]** | **0.00** |
| **Interventions (boolean for 1st 24 h)** |  |  |  |  |  |  |
| Mechanical ventilation use (YES) | **232 (74.60%)** | **12990 (47.17%)** | **4887 (63.88%)** | **<0.001** | **[0.586, 0.234]** | **0.00** |
| Vasopressor use (YES) | **200 (64.31%)** | **11293 (41.01%)** | **3457 (45.19%)** | **<0.001** | **[0.480, 0.391]** | **0.00** |
| Sedative use (YES) | **207 (66.56%)** | **12836 (46.61%)** | **4640 (60.65%)** | **<0.001** | **[0.411, 0.123]** | **0.00** |
| Albumin use (YES) | **50 (16.08%)** | **4144 (15.05%)** | **836 (10.93%)** | **<0.001** | **[0.028, 0.151]** | **0.00** |
| **Comorbidities (boolean)** |  |  |  |  |  |  |
| HF (YES) | **117 (37.62%)** | **9355 (33.97%)** | **1949 (25.48%)** | **<0.001** | **[0.079, 0.266]** | **0.03** |
| AFIB (YES) | **83 (26.69%)** | **4568 (16.59%)** | **1059 (13.84%)** | **<0.001** | **[0.249, 0.326]** | **0.03** |
| Renal (YES) | **94 (30.23%)** | **7730 (28.07%)** | **1516 (19.82%)** | **<0.001** | **[0.049, 0.244]** | **0.03** |
| Liver (YES) | **20 (6.43%)** | **2776 (10.08%)** | **493 (6.44%)** | **<0.001** | **[0.132, <0.001]** | **0.03** |
| COPD (YES) | **51 (16.40%)** | **4704 (17.08%)** | **921 (12.04%)** | **<0.001** | **[0.017, 0.126]** | **0.03** |
| CAD (YES) | **108 (34.73%)** | **8979 (32.61%)** | **1801 (23.54%)** | **<0.001** | **[0.047, 0.250]** | **0.03** |
| Stroke (YES) | **31 (9.97%)** | **2853 (10.36%)** | **1059 (13.84%)** | **<0.001** | **[0.012, 0.119]** | **0.03** |
| Malignancy (YES) | **30 (9.65%)** | **4566 (16.58%)** | **1191 (15.57%)** | **<0.001** | **[0.206, 0.178]** | **0.03** |
| **Vital signs (1st 24 h)** |  |  |  |  |  |  |
| MAP | **80.00 [68.00, 93.50]** | **80.00 [69.00, 92.00]** | **81.00 [70.00, 94.00]** | **<0.001** | **[0.006, 0.047]** | **0.00** |
| Heart rate | **83.00 [69.00, 97.00]** | **87.00 [76.00, 102.00]** | **97.00 [82.00, 113.00]** | **<0.001** | **[0.277, 0.678]** | **0.00** |
| **Laboratory tests (1st 24 h)** |  |  |  |  |  |  |
| WBC | **13.40 [8.45, 18.85]** | **11.40 [7.90, 16.10]** | **11.85 [8.10, 16.62]** | **<0.001** | **[0.169, 0.116]** | **0.03** |
| Hemoglobin | **10.60 [8.95, 12.80]** | **10.00 [8.50, 11.60]** | **10.40 [8.80, 12.10]** | **<0.001** | **[0.298, 0.159]** | **0.02** |
| Platelet | **202.00 [140.00, 269.00]** | **183.00 [125.00, 258.00]** | **189.00 [130.00, 262.00]** | **<0.001** | **[0.091, 0.053]** | **0.08** |
| Sodium | **138.00 [135.00, 141.00]** | **138.00 [135.00, 141.00]** | **138.00 [135.00, 141.00]** | **<0.001** | **[0.052, 0.033]** | **0.01** |
| Potassium | **4.30 [3.80, 4.80]** | **4.20 [3.80, 4.70]** | **4.10 [3.70, 4.60]** | **<0.001** | **[0.093, 0.249]** | **0.01** |
| Bicarbonate | **20.00 [16.00, 23.00]** | **23.00 [20.00, 25.00]** | **22.00 [20.00, 25.00]** | **<0.001** | **[0.490, 0.483]** | **0.02** |
| Chloride | **105.00 [100.00, 108.00]** | **104.00 [99.00, 108.00]** | **104.00 [100.00, 108.00]** | **<0.001** | **[0.095, 0.026]** | **0.02** |
| BUN | **27.00 [18.00, 47.50]** | **23.00 [15.00, 41.00]** | **20.00 [13.00, 32.00]** | **<0.001** | **[0.216, 0.469]** | **0.03** |
| Lactate | **2.90 [1.80, 5.30]** | **1.80 [1.20, 2.60]** | **1.80 [1.20, 2.70]** | **<0.001** | **[0.651, 0.656]** | **11.61** |
| Creatinine | **1.40 [0.90, 2.70]** | **1.10 [0.80, 1.90]** | **1.00 [0.80, 1.60]** | **<0.001** | **[0.249, 0.374]** | **0.02** |
| pH | **7.28 [7.19, 7.37]** | **7.37 [7.31, 7.42]** | **7.37 [7.31, 7.43]** | **<0.001** | **[0.751, 0.760]** | **16.70** |
| PO2 | **121.00 [60.00, 245.00]** | **124.00 [76.00, 254.00]** | **119.00 [78.00, 211.00]** | **<0.001** | **[0.059, 0.049]** | **37.90** |
| PCO2 | **40.00 [34.00, 51.00]** | **41.00 [35.00, 49.00]** | **41.00 [35.00, 48.00]** | **<0.01** | **[0.010, 0.077]** | **39.34** |
| **Outcomes (boolean)** |  |  |  |  |  |  |
| 28-day mortality (Death) | **169 (54.34%)** | **5845 (21.23%)** | **1475 (19.28%)** | **<0.001** | **[0.727, 0.780]** | **0.00** |
| ICU mortality (Death) | **138 (44.37%)** | **3186 (11.57%)** | **986 (12.89%)** | **<0.001** | **[0.785, 0.743]** | **0.00** |
| In-hospital mortality (Death) | **153 (49.20%)** | **4773 (17.33%)** | **1336 (17.46%)** | **<0.001** | **[0.719, 0.715]** | **0.00** |
| **Length of Stay (days)** |  |  |  |  |  |  |
| ICU length of Stay | **3.74 [2.17, 7.08]** | **3.19 [1.86, 6.56]** | **4.30 [2.30, 9.01]** | **<0.001** | **[0.106, 0.093]** | **0.00** |
| Hospital length of Stay | **6.23 [3.42, 11.82]** | **8.33 [5.09, 15.33]** | **10.38 [6.02, 18.55]** | **<0.001** | **[0.174, 0.310]** | **0.00** |
| Values are presented as mean (standard deviation) or median [Q1, Q3] for continuous variables and number (percentage) for categorical variables. Variables in bold have p-value < 0.05. | | | | | | |

Supplementary Table 2. Unadjusted log-rank test for 28-day mortality of original cohort

| Group | HR^1^ | 95% CI^1^ | p-value |
| --- | --- | --- | --- |
| <36℃ vs 36-38℃ | 0.27 | 0.20, 0.36 | <0.001 |
| <36℃ vs >38℃ | 0.25 | 0.18, 0.33 | <0.001 |
| 36℃-38℃ vs >38℃ | 0.90 | 0.86, 0.96 | <0.001 |
| ^1^HR = Hazard Ratio, CI = Confidence Interval; | | | |

Supplementary Table 3. Unadjusted log-rank test for ICU mortality of original cohort

| Group | HR^1^ | 95% CI^1^ | p-value |
| --- | --- | --- | --- |
| <36℃ vs 36-38℃ | 0.30 | 0.22, 0.41 | <0.001 |
| <36℃ vs >38℃ | 0.26 | 0.19, 0.35 | <0.001 |
| 36℃-38℃ vs >38℃ | 0.84 | 0.79, 0.90 | <0.001 |
| ^1^HR = Hazard Ratio, CI = Confidence Interval; | | | |

***Supplementary Table 4. Unadjusted log-rank test for In-hospital mortality of original cohort***

| Group | HR^1^ | 95% CI^1^ | p-value |
| --- | --- | --- | --- |
| <36℃ vs 36-38℃ | 0.29 | 0.21, 0.39 | <0.001 |
| <36℃ vs >38℃ | 0.25 | 0.18, 0.34 | <0.001 |
| 36℃-38℃ vs >38℃ | 0.86 | 0.81, 0.91 | <0.001 |
| ^1^HR = Hazard Ratio, CI = Confidence Interval; | | | |

Supplementary Table 5. Standardized mean difference (SMD) of covariates before and after propensity score matching of cohort 1

| Characteristic | Before matcing | After matcing |
| --- | --- | --- |
| SMD ≤ 0.1 | 16 | 29 |
| SMD > 0.1 | 16 | 3 |
| Total number of covariates | 32 | 32 |

Supplementary Table 6. Standardized mean difference (SMD) of covariates before and after propensity score matching of cohort 2

| Characteristic | Before matcing | After matcing |
| --- | --- | --- |
| SMD ≤ 0.1 | 7 | 31 |
| SMD > 0.1 | 25 | 1 |
| Total number of covariates | 32 | 32 |

Supplementary Table 7. Standardized mean difference (SMD) of covariates before and after propensity score matching of cohort 3

| Characteristic | Before matcing | After matcing |
| --- | --- | --- |
| SMD ≤ 0.1 | 13 | 32 |
| SMD > 0.1 | 19 | 0 |
| Total number of covariates | 32 | 32 |
